# Prognostic value of admission electrocardiographic findings in non‐ST‐segment elevation myocardial infarction

**DOI:** 10.1002/clc.23349

**Published:** 2020-03-03

**Authors:** Peng‐fei Chen, Liang Tang, Jun‐yu Pei, Jun‐lin Yi, Zhen‐hua Xing, Zhen‐fei Fang, Sheng‐hua Zhou, Xin‐qun Hu

**Affiliations:** ^1^ Department of Cardiology Second Xiangya Hospital of Central South University Changsha China

**Keywords:** electrocardiogram, invasive management, non‐ST‐segment elevation myocardial infarction, prognosis

## Abstract

**Background:**

Admission electrocardiographic (ECG) findings of non‐ST‐segment elevation myocardial infarction (NSTEMI) include transient ST‐segment elevation (TSTE), ST‐segment depression (STD), T‐wave inversion (TWI), and no ischemic changes (NIC).

**Hypothesis:**

This study aimed to assess the prognostic value of qualitative ECG findings at presentation and to clarify the influence of invasive treatment on the prognostic value of admission ECG findings.

**Methods:**

We analyzed the Acute Coronary Syndrome Quality Improvement in Kerala (ACS QUIK) study post hoc. NSTEMI patients were included and classified into four groups per ECG findings. Study endpoints were in‐hospital and 30‐day mortality rates and major adverse events (MAE). We performed multivariate logistic regression, adjusting for covariates in the Global Registry of Acute Coronary Events risk model, with subset analyses of patients treated with or without invasive management.

**Results:**

STD patients had significantly higher in‐hospital and 30‐day mortality rates/MAE than TWI patients, which had lower in‐hospital mortality rate/MAE than the NIC group. TSTE patients had intermediate outcomes. In multivariate logistic regression using the TWI group as the reference, STD and NIC remained independently associated with worse outcomes. Subset analysis showed prognostic value of admission ECG in non‐invasively managed but not in invasively managed patients.

**Conclusions:**

STD was associated with adverse outcomes, TWI with benign prognoses. NIC should not be taken to indicate low risk. Qualitative analysis of admission ECG is suitable for rapid risk stratification of NSTMI patients at presentation. However, it may not be predictive of short‐term outcomes of NSTEMI patients after invasive management.

## 1 INTRODUCTION

The electrocardiogram (ECG) is the most accessible and widely used diagnostic tool when patients are suspected to have acute coronary syndrome (ACS).^1^ Admission ECG findings may also provide prognostic information and rapid risk assessment in ACS patients,^2^, ^3^ especially those with non‐ST‐segment elevation myocardial infarction (NSTEMI), a diagnosis applied to a heterogeneous group of patients with different clinical characteristics and outcomes. ECG findings of NSTEMI at presentation show several variations, including transient ST‐segment elevation (TSTE), ST‐segment depression (STD), T‐wave inversion (TWI), and no ischemic changes (NIC). Previous studies have shown that the presence of STD is associated with an increased risk of short‐ and long‐term cardiovascular (CV) events,^4^, ^5^ but studies of TWI have shown inconsistent results.^6^, ^7^, ^8^, ^9^, ^10^, ^11^ Limited data are available on patients with TSTE and NIC. Risk stratification remains crucial to selecting appropriate management strategies for NSTEMI patients. Therefore, systematically investigating these four ECG subgroups would help clinicians better risk‐stratify patients and yield additional insight into the selection of appropriate treatment. Furthermore, current guidelines for NSTEMI patients recommend invasive reperfusion to improve clinical outcomes,^12^, ^13^ but the effect of invasive management on the prognostic value of admission ECG data in contemporary practice has not been studied. It is still unclear whether the prognostic value of admission ECG findings differs between patients with or without invasive management.

In this study, we performed a retrospective analysis of NSTE‐ACS patients in the Acute Coronary Syndrome Quality Improvement in Kerala (ACS QUIK) trial, comparing patient characteristics, treatments, and outcomes between four ECG subgroups with the aim of systematically assessing the prognostic value of qualitative ECG findings at presentation. We also performed a subset analysis of patients with or without invasive reperfusion in order to clarify the influence of such treatment on the prognostic value of admission ECG findings.

## 2 METHODS

### 2.1 Study population

We obtained ACS QUIK study data from the Biologic Specimen and Data Repository Information Coordinating Center (BioLINCC; National Heart, Lung, and Blood Institute, Bethesda, Maryland). The rationale and design have been previously described.^14^, ^15^ Briefly, 63 hospitals in Kerala were recruited and randomized to receive a quality improvement tool kit intervention from November 10, 2014 to November 9, 2016. Patients were eligible to participate if they presented with either NSTEMI or ST‐segment elevation myocardial infarction (STEMI), based on the Third Universal Definition of Myocardial Infarction. Written informed consent was obtained from all participants or their proxies. Data were collected according to the 2013 American College of Cardiology/American Heart Association “Key data elements and definitions for measuring the clinical management and outcomes key of patients with acute coronary syndromes and coronary artery disease.”^16^ We obtained data for patients 30 days post hospitalization. For the present analysis, we included all NSTEMI patients with available ECG data at admission.

### 2.2 ECG classification

The qualitative category of ECG finding for each patient was indicated by a checkmark in the appropriate box on the ACS QUIK study case report form. We divided patients into four groups based on ECG findings at presentation: TSTE, STD, TWI, and NIC. TSTE was defined as new or presumed‐new ST‐segment elevation lasting <20 minutes at the J point in two contiguous leads; with the cutpoints ≥0.1 mV in all leads other than V2‐V3, where the following cutpoints applied: ≥0.2 mV in men age ≥ 40 years, ≥0.25 mV in men age < 40 years, or ≥0.15 mV in women. STD was defined as new or presumed‐new horizontal or downsloping ST depression ≥0.05 mV in two contiguous leads below the isoelectric line on the ECG. TWI was defined as new or presumed‐new TWI of ≥0.1 mV in two contiguous leads with prominent R wave or R/S ratio > 1. If the ECG indicated TSTE, no matter whether other findings (STD and TWI) were present, the patient was assigned to the TSTE group. If the ECG revealed STD and TWI (but not TSTE), the patient was put into the STD group. Patients whose ECGs showed only TWI were classified into the TWI group. The ECG was considered to show NIC if the admission ECG had not revealed STD, TSTE, or TWI.

### 2.3 Study endpoint

Study endpoints were in‐hospital and 30‐day mortality rates and major adverse events (MAE), the latter defined as the composite of death, reinfarction, stroke, and major bleeding events (MBEs). MBEs were defined as intracerebral hemorrhage or bleeding resulting in substantial hemodynamic compromise and requiring treatment by Global Utilization of Streptokinase and Tissue Plasminogen Activator for Occluded Coronary Arteries (GUSTO) criteria. Follow‐up data were collected at each site, either through in‐person visits or by telephone. If a site was unable to reach a participant after three attempts, then that participant was considered lost to follow‐up. Of all patients, 99% completed 30 days of follow‐up.

### 2.4 Statistical analysis

All statistical tests were performed using SPSS software version 20.0 (SPSS Inc., Chicago, Illinois). We compared baseline characteristics, treatments, and clinical outcomes across the four ECG categories, conducting both four‐way and pairwise comparisons. Continuous variables were presented as the median and interquartile range and compared using Kruskal‐Wallis tests. Categorical data were expressed as percentages and compared by chi‐square test or Fisher's exact test. To determine the independent prognostic significance of admission ECG findings, we performed a multivariate logistic‐regression analysis using the TWI group as the reference and adjusting for components of the Global Registry of Acute Coronary Events (GRACE) risk models, which included age, heart rate, systolic blood pressure, Killip class, and presentation with cardiac arrest. Serum creatinine (sCr) levels were not included because these data were not available for about one‐third of patients. We calculated the adjusted odds ratios (ORs) by multivariate analysis. The subset analysis was performed by comparing outcomes in the four groups between patients with invasive management and patients without. Patients undergoing coronary angiography (CAG) or percutaneous coronary intervention (PCI) were considered as receiving invasive management. Adjusted ORs for patients with or without invasive management were also calculated by multivariate analysis.

## 3 RESULTS

### 3.1 Distribution of ECG findings

A total of 21 374 patients were enrolled in the ACS QUIK trial, 7684 of whom had NSTEMI. We included the NSTEMI patients in the present analysis and grouped them by ECG findings at presentation: TSTE (n = 197, 2.6%), STD (n = 3603, 46.9%), TWI (n = 2384, 31%), and NIC (n = 1500, 19.5%). In the TSTE group, 117 patients (1.5%) had isolated TSTE, 35 (0.5%) had STD, 44 (0.6%) had TWI, and only 1 had all 3 ischemic ECG changes. In the STD group, 2868 patients (37.3%) had isolated STD, whereas 735 (9.6%) had both STD and TWI. The distribution of ECG findings at presentation is shown in Figure S1.

### 3.2 Baseline characteristics

Baseline characteristics differed among the four ECG groups. Patients with TSTE had the highest initial troponin levels; for other baseline characteristics, TSTE patients were similar to TWI patients and more likely to be current tobacco users than NIC patients. Patients with STD trended toward older age, more‐rapid heart rate, and more comorbidities, such as hypertension and diabetes when compared with TWI patients. The STD group was also more likely to be female, current tobacco users, presenting with heart failure and having higher levels of initial troponin compared with the NIC group. Overall, patients with TSTE or STD had more risk characteristics than did those with TWI or NIC. When compared with the STD group, the TSTE group was younger, more frequently male, and more‐frequent smokers, but with higher initial troponin levels. Patients with NIC had a higher incidence of hypertension and diabetes but a lower incidence of current smoking than patients with TWI. Baseline characteristics of the four groups are summarized in Table S1.

### 3.3 Medication and procedure

Frequencies of pre‐hospital and in‐hospital antiplatelet drug use were similar across all four groups. The exception was for glycoprotein IIb/IIIa inhibitors, which were most frequently used in patients with TSTE, followed by those with TWI and NIC, and least used in patients with STD (pairwise *P* < .08 except for TWI vs NIC). Anticoagulants were less used in the NIC group than in the other three groups (pairwise *P* < .08). The use of beta‐blockers was more common in patients with TSTE and TWI than in those with STD and NIC (pairwise *P* < .08). CAG and PCI were both performed mostly in patients with TSTE, least in patients with STD. The four groups did not significantly differ in frequency of coronary‐artery bypass grafting performance. Overall, patients with TSTE were more likely to receive optimal medication and invasive management, followed by patients with TWI and NIC; those with STD were least likely. Detailed medication and procedure data are presented in Table S2.

### 3.4 In‐hospital and 30‐day outcomes

The incidence of mortality and MAE in the hospital or after 30 days of follow‐up was highest in the STD group, followed by the NIC and TSTE groups, and lowest in the TWI group. However, only two of these differences were statistically significant: STD vs TWI and NIC vs TWI. When compared with the TWI group, patients with STD had significantly higher in‐hospital and 30‐day mortality rates and MAE, while those with NIC had significantly higher in‐hospital mortality rates and MAE. Detailed outcome data are shown in Table 1. In multivariate analysis, with the TWI group as the reference, STD and NIC were independently associated with worse outcomes. Results of multivariate analysis are listed in Table 2.

**Table 1:** In‐hospital and 30‐day clinical outcomes in NSTEMI patients classified by admission electrocardiographic findings

	TSTE(n = 197)	STD(n = 3603)	TWI(n = 2384)	NIC(n = 1500)	*P* value(four‐way)
In‐hospital outcomes, n (%)					
All‐cause mortality	2 (1)	90 (2.5)	22 (0.9)	32 (2.1)	<.001§#
MAE	3 (1.5)	123 (3.4)	37 (1.6)	42 (2.8)	<.001§#
Major GUSTO bleeding	0 (0)	7 (0.2)	1 (0.04)	0 (0)	.14
Stroke	0 (0)	23 (0.6)	7 (0.3)	6 (0.4)	.181
Reinfarction	2 (1)	23 (0.6)	10 (0.4)	4 (0.3)	.229
30‐day outcomes, n (%)					
All‐cause mortality	6 (3.1)	162 (4.6)	60 (2.6)	52 (3.5)	.001§
MAE	9 (4.7)	214 (6)	88 (3.7)	71 (4.8)	.002§
Major GUSTO bleeding	0 (0)	9 (0.3)	3 (0.1)	1 (0.07)	.558
Stroke	0 (0)	27 (0.8)	14 (0.6)	10 (0.7)	.594
Reinfarction	4 (2.1)	43 (1.2)	23 (0.9)	10 (0.7)	.179

*Note*: For pairwise comparisons: §*P* < .008, STD group vs TWI group; #*P* < .008, TWI group vs NIC group.

Abbreviations: GUSTO, Global Utilization of Streptokinase and Tissue Plasminogen Activator for Occluded Coronary Arteries; MAE, major adverse events; NIC, no ischemic changes; STD, ST‐segment depression; TSTE, transient ST‐segment elevation; TWI, T‐wave inversion.

**Table 2:** Multivariate logistic‐regression analysis of admission electrocardiographic findings

	In‐hospital mortality		In‐hospital MAE		30‐day mortality		30‐day MAE	
	Adjusted OR (95% CI)	*P*	Adjusted OR (95% CI)	*P*	Adjusted OR (95% CI)	*P*	Adjusted OR (95% CI)	*P*
TSTE	0.139‐3.341	.636	0.158‐2.304	.46	0.371‐2.407	.906	0.489‐2.252	.901
STD	1.541‐4.139	<.001	1.408‐3.059	<.001	1.199‐2.246	.002	1.17‐1.978	.002
NIC	1.093‐3.475	.024	0.978‐2.506	.062	0.799‐1.76	.398	0.829‐1.612	.394
TWI	Reference		Reference		Reference		Reference	

*Note*: Adjusting for age, heart rate, systolic blood pressure, Killip class, and presentation with cardiac arrest.

Abbreviations: MAE, major adverse events; NIC, no ischemic changes; OR, odds ratio; STD, ST‐segment depression; TSTE, transient ST‐segment elevation; TWI, T‐wave inversion.

### 3.5 Outcomes in patients with or without invasive management

**Figure 1:**
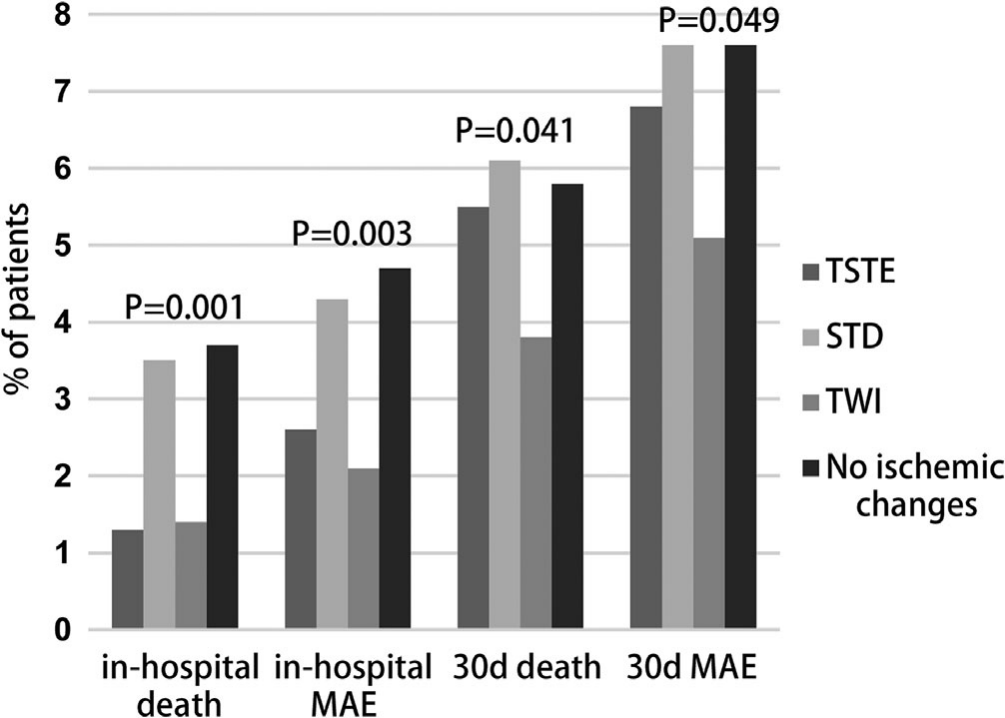
In‐hospital and 30‐day outcomes across ECG subgroups in NSTEMI patients without invasive management

**Figure 2:**
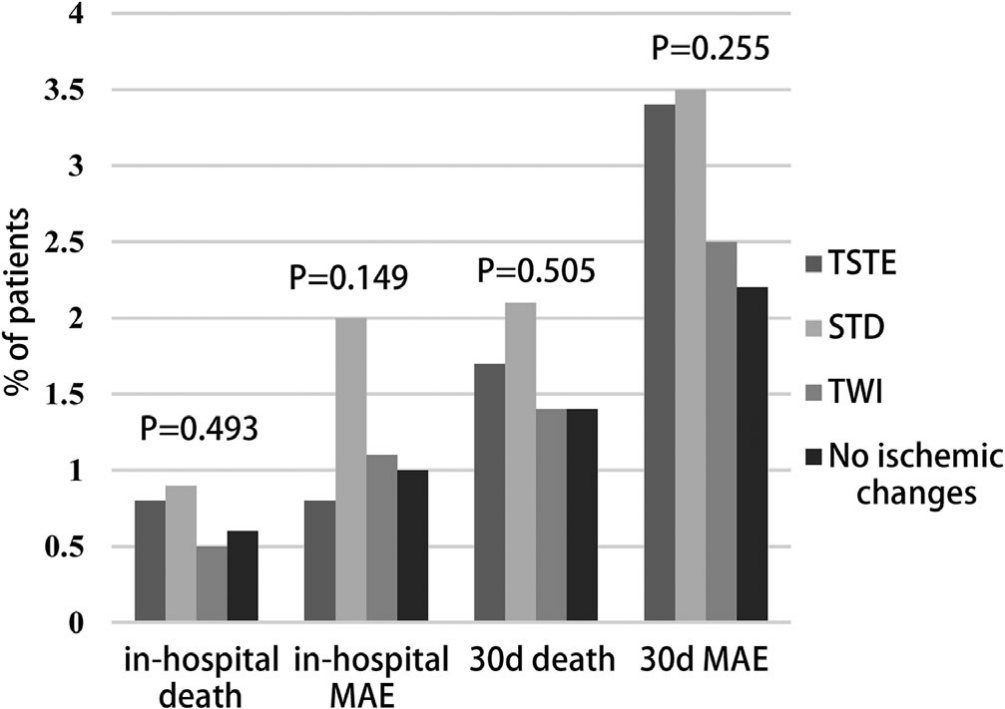
In‐hospital and 30‐day outcomes across ECG subgroups in NSTEMI patients with invasive management

Of patients who were not invasively managed, the STD group had the highest in‐hospital or 30‐day mortality rate and MAE, while the TWI group had the lowest in‐hospital MAE and 30‐day mortality rate and MAE (Figure 1). Patients with STD had significantly higher in‐hospital and 30‐day mortality rate and MAE than patients with TWI. Patients with NIC also had significantly higher in‐hospital mortality rate and MAE than did patients with TWI. In multivariate analysis, STD and NIC were still independently associated with worse outcomes. In invasively managed patients, in‐hospital and 30‐day mortality rates and MAE were similar across all four groups (Figure 2). We did not observe admission ECG findings to have prognostic value in patients with invasive management. Results of multivariate analysis are listed in Table 3.

**Table 3:** Multivariate logistic‐regression analysis of admission electrocardiographic findings in patients with or without invasive management

	In‐hospital mortality		In‐hospital MAE		30‐day mortality		30‐day MAE	
	Adjusted OR (95% CI)	*P*	Adjusted OR (95% CI)	*P*	Adjusted OR (95% CI)	*P*	Adjusted OR (95% CI)	*P*
Patients with invasive management								
TSTE vs TWI	0.154‐13.521	.749	0.095‐6.024	.792	0.206‐4.855	1.0	0.437‐3.907	.632
STD vs TWI	0.454‐3.729	.624	0.794‐3.165	.192	0.607‐2.188	.665	0.756‐1.95	.422
NIC vs TWI	0.288‐3.66	.967	0.309‐2.007	.617	0.408‐2.026	.816	0.413‐1.437	.412
Patients without invasive management								
TSTE vs TWI	0.032‐3.516	.36	0.097‐3.257	.519	0.268‐3.042	.87	0.321‐2.754	.911
STD vs TWI	1.62‐5.227	<.001	1.409‐3.685	.001	1.156‐2.413	.006	1.132‐2.153	.007
NIC vs TWI	1.213‐4.678	.012	1.17‐3.629	.012	0.838‐2.109	.226	0.938‐2.097	.099

*Note*: Adjusting for age, heart rate, systolic blood pressure, Killip class, and presentation with cardiac arrest.

Abbreviations: MAE, major adverse events; NIC, no ischemic changes; OR, odds ratio; STD, ST‐segment depression; TSTE, transient ST‐segment elevation; TWI, T‐wave inversion.

## 4 DISCUSSION

In the present analysis of the patient‐pooled data set from the ACS QUIK trail, we found that in patients presenting with NSTEMI, STD was the most common finding on admission ECG, followed by TWI, NIC, and TSTE. Different associations were revealed between clinical outcomes and the four ECG categories. However, in subset analysis, we observed these associations only in patients who did not receive invasive management, not in those who did receive such management.

The prognostic significance of STD has been well established by previous studies.^4^, ^5^ It is recognized as a powerful predictor of adverse outcomes and has been incorporated into various frequently used risk models for ACS.^17^, ^18^ Indeed, in the present study, STD was the most frequent finding on admission ECG, constituting a distinct group of patients with more risk characters and worse clinical outcomes. However, when we classified patients by reperfusion management, we found that STD had outcome‐predictive value only in patients who were not invasively managed. For patients treated with invasive therapy, in‐hospital and 30‐day outcomes were similar across all four ECG categories. Thus, qualitative analysis of STD on admission ECG may not provide prognostic information for short‐term outcomes of NSTEMI patients who received invasive management. The underlying reason is unclear, but we suspect that the presence of STD in ACS patients indicates a high ischemic burden, which may be the substrate for worse outcomes and can be resolved by invasive management. Interestingly, despite being a high‐risk population, these patients were less likely to be treated with CAG or revascularization. Previous studies have also shown an inverse relationship between risk status and rate of invasive management,^19^, ^20^ notwithstanding that invasive reperfusion has been proven beneficial.^21^ Therefore, the current practice patterns for this high‐risk group probably represent an opportunity for improvement.

The prognostic value of TWI has also been previously explored using data from other randomized clinical trials. Savonitto et al studied admission ECGs from the GUSTO‐IIB trial and found that the isolated TWI group had lower incidence of death and reinfarction at both 30 days' and 6 months' follow‐up.^2^ However, patients with NIC on their presenting ECGs were not included in the GUSTO‐IIB trial; therefore, the sub‐study did not have a group without ischemic changes. Our results provided further evidence of the relatively benign prognosis of isolated TWI by directly comparing its outcomes with those of NIC. We found that even compared with NIC, isolated TWI was still associated with a lower adjusted in‐hospital mortality rate. This is compatible with a previous analysis from a national CV registry data with a large population,^6^ even though some relatively small studies have reported isolated TWI not to be an independent prognosticator.^7^, ^8^ Yet other studies have even found that TWI combined with STD was linked to poor outcomes.^9^, ^10^, ^11^ However, the factors accounting for adverse outcomes might be STD and other associated prognosticators but not TWI itself. A recent study found a relation between TWI and the presence of myocardial edema, a relatively light and reversible injury in patients with ACS.^22^ TWI may represent a lower ischemic burden, therefore having relatively benign outcomes. Note that in the present study, the predictive value of isolated TWI, like that of STD, was also observed only in patients without invasive management.

Although previous studies have confirmed that patients who present with ST‐segment deviation and/or TWI have a greater likelihood of having ACS, and those presenting with NIC have a lower likelihood of having ACS,^12^, ^13^ a “negative” ECG is not uncommon in ACS patients. In our study, those with NIC constituted 19.5% of patients with NSTEMI. In both Patel's and Cannon's studies, nearly 60% of NSTEMI patients presented with NIC.^3^, ^6^ More importantly, a “negative” initial ECG should not be taken as an indicator of a favorable outcome. Our study showed that in the total study population, patients presenting with NIC on the admission ECG had the second‐highest incidence of mortality and MAE both in the hospital and after 30 days' follow‐up. Even after adjustment for established GRACE risk score prognosticators, the NIC group still had a significantly higher in‐hospital mortality rate than the TWI group. Therefore, in current practice, patients with no ischemic ECG changes should not be taken for granted as low‐risk; more attention should be paid to the management of such patients.

Although our study showed that TSTE was the least frequent finding on the admission ECG and had no prognostic value for short‐term outcome, our investigation into this finding still bears important clinical implications, since it is unclear at present whether TSTE myocardial infarction (TSTEMI) should be considered a variant of STEMI or a subgroup of NSTEMI. Previous studies have revealed that TSTEMI is associated with less ischemic damage and better short‐ or long‐term outcomes than STEMI.^23^, ^24^ In this study, we explored TSTEMI in the context of NSTEMI and found that it had similar in‐hospital and 30‐day outcomes as the other three categories, regardless of whether patients received invasive management. These results corroborated data from several observational studies showing no differences in outcome during hospitalization or short‐term follow‐up.^6^, ^25^ From this perspective, TSTEMI seems to behave more like NSTEMI than STEMI. However, Badings et al and Blondheim et al have reported favorable long‐term outcomes in TSTEMI vs NSTEMI patients.^24^, ^25^ In addition, Lemkes et al recently performed a randomized study to assess the effect of an immediate vs a delayed invasive strategy in patients with TSTEMI and found no difference in myocardial‐infarct size or 30‐day outcome between these two strategies.^26^ Badings et al also found that in patients with high‐risk NSTE‐ACS, early angiography and revascularization in TSTE patients were not superior to later treatment.^25^ Thus, long‐term outcomes and management of TSTEMI might be somewhat different from those of NSTEMI. It is therefore more reasonable to treat TSTEMI as a new classification.

There are some limitations that we should acknowledge. First, we did not perform quantitative ECG analysis. The quantitative characteristics of admission ECG, such as degree of ST‐segment deviation or number of leads involved, are also reported to be independently associated with clinical outcomes.^11^, ^27^, ^28^ However, there is controversy over the usefulness of quantitative ECG analysis, as it requires more expertise and is more time consuming. Previous studies have shown that such analysis may not provide additional prognostic value over qualitative ECG analysis.^29^, ^30^ Second, the short‐term follow‐up limits the interpretation of results. The association between admission ECG findings and long‐term outcomes might be different: Mueller et al found that STD and TWI were still important predictors for long‐term outcome in NSTE‐ACS patients who received early invasive management.^31^ Therefore, the prognostic value of admission ECG findings for long‐term outcomes still need further investigation. Third, sCr levels were not included in the multivariate logistic‐regression analysis because sCr data were available for only 66.1% of patients. However, we did analyze this available data and found that sCr levels were similar across the four ECG groups. The difference in prognosis between the four ECG categories was therefore not affected by sCr level.

## 5 CONCLUSION

The presence of STD on admission ECG was associated with adverse outcomes, while that of TWI was linked to benign short‐term prognoses. A lack of ischemic ECG changes should not be taken as an indicator of low risk. TSTE did not show any prognostic value for short‐term outcomes. Therefore, qualitative analysis of admission ECG is suitable for rapid risk stratification of NSTMI patients at presentation. However, it may not provide predictive information of short‐term outcomes for NSTEMI patients after they receive invasive management.

## CONFLICT OF INTEREST

The authors declare no potential conflict of interests.

## Supporting information


**Table S1** Baseline characteristics in NSTEMI patients classified by admission electrocardiographic findings.
**Table S2.** Medication and procedural data in NSTEMI patients classified by admission electrocardiographic findings.

**Figure S1**. Distribution of admission electrocardiographic findings.
