# Supplementary material for: Prognostic value of admission electrocardiographic findings in non‐ST‐segment elevation myocardial infarction
Source: Clin Cardiol. 2020 Mar 3;43(6):574–80. doi: 10.1002/clc.23349 (PMC7299002; doi:10.1002/clc.23349)
Supplement: Supplementary file 1 — Table S1 Baseline characteristics in NSTEMI patients classified by admission electrocardiographic findings. Table S2. Medication and procedural data in NSTEMI patients classified by admission electrocardiographic findings. [file CLC-43-574-s001.docx]

Online Table 1. Baseline characteristics in NSTEMI patients classified by admission electrocardiographic findings.

|  | **TSTE**  **(n=197)** | **STD**  **(n=3603)** | **TWI**  **(n=2384)** | **NIC**  **(n=1500)** | **P Value**  **（four way）** |
| --- | --- | --- | --- | --- | --- |
| **Demographics** |  |  |  |  |  |
| Age (years) | 60 (51-69) | 63 (55-72) | 61 (53-70) | 63 (54-71) | ＜0.001*§ |
| Male, n (%) | 155 (78.7) | 2439 (67.7) | 1667 (69.9) | 1078 (71.9) | 0.001*¶ |
| **History and risk factors, n(%)** |  |  |  |  |  |
| Current tobacco use | 57 (28.9) | 864 (24) | 596 (25) | 303 (20.2) | 0.001‡¶# |
| Diabetes mellitus | 99 (50.3) | 1923 (53.4) | 1142 (47.9) | 843 (56.2) | ＜0.001§# |
| Hypertension | 104 (52.8) | 2185 (60.6) | 1314 (55.1) | 925 (61.7) | ＜0.001§# |
| Peripheral arterial disease | 0 (0) | 62 (1.7) | 33 (1.4) | 16 (1.1) | 0.094 |
| Prior TIA/Stroke | 3 (1.5) | 101 (2.8) | 78 (3.3) | 43 (2.9) | 0.462 |
| Dialysis | 0 (0) | 31 (0.9) | 15 (0.6) | 20 (1.3) | 0.067 |
| **Cardiac status at admission** |  |  |  |  |  |
| SBP (mm Hg) | 140 (130-160) | 140 (122-160) | 140 (122-160) | 140 (120-160) | ＜0.001¶# |
| Heart rate (beats/min) | 80 (71-92) | 80 (70-95) | 78.5 (68-90) | 80 (70-96) | ＜0.001§# |
| Heart failure, n(%) | 22 (11.2) | 418 (11.6) | 237 (9.9) | 133 (8.9) | 0.021¶ |
| Cardiogenic Shock, n(%) | 4 (2) | 35 (1) | 18 (0.8) | 18 (1.2) | 0.236 |
| Cardiac Arrest, n(%) | 3 (1.5) | 20 (0.5) | 18 (0.8) | 13 (0.9) | 0.296 |
| Killip Class III-IV, n (%) | 19 (9.6) | 402 (11.2) | 227 (9.5) | 199 (13.3) | 0.003# |
| LVEF (%) | 55 (47.5-62) | 55 (50-60) | 55 (49-60) | 56 (50-60) | 0.088 |
| **Laboratory tests** |  |  |  |  |  |
| Initial troponin (ng/ml) | 2.4 (0.36-8.96) | 1.09 (0.29-3.91) | 0.97 (0.24-3.57) | 0.83 (0.22-2.80) | ＜0.001*†‡¶ |
| LDL-C (mg/dl) | 117 (90.5-144) | 117 (89-144) | 118 (90-148) | 104 (78-136) | ＜0.001¶# |
| HDL-C (mg/dl) | 42 (35-47) | 42 (36-48) | 41 (34.5-48) | 40 (33-48) | ＜0.001¶ |
| Serum creatinine (mg/dl)^a^ | 1.1 (0.915-1.4) | 1.1 (0.9-1.32) | 1.1 (0.9-1.3) | 1.1 (0.9-1.3) | 0.093 |
| Fasting Glucose (mg/dl) | 126.5 (105.25-180.75) | 128 (100-180) | 123 (98-170) | 133 (105-183) | ＜0.001¶# |

Data are expressed as median (interquartile range), or as number (percentage). ^a^ Serum creatinine data were only available in 66.1% patients. For pairwise comparisons: *P <0.008, TSTE group vs STD group; †P <0.008, TSTE group vs TWI group; ‡P <0.008, TSTE group vs NIC group; §P <0.008, STD group vs TWI group; ¶P <0.008, STD group vs NIC group; #P <0.008, TWI group vs NIC group. TSTE = transient ST-segment elevation; STD = ST-segment depression; TWI = T-wave inversion; NIC = no ischemic changes; TIA = transient ischemic attack; SBP = systolic blood pressure; LVEF = left ventricular ejection fraction; LDL-C = low-density lipoprotein cholesterol; HDL-C = high-density lipoprotein cholesterol.
