# Supplementary figures and images for: Prognostic value of admission electrocardiographic findings in non‐ST‐segment elevation myocardial infarction
Source: Clin Cardiol. 2020 Mar 3;43(6):574–80. doi: 10.1002/clc.23349 (PMC7299002; doi:10.1002/clc.23349)

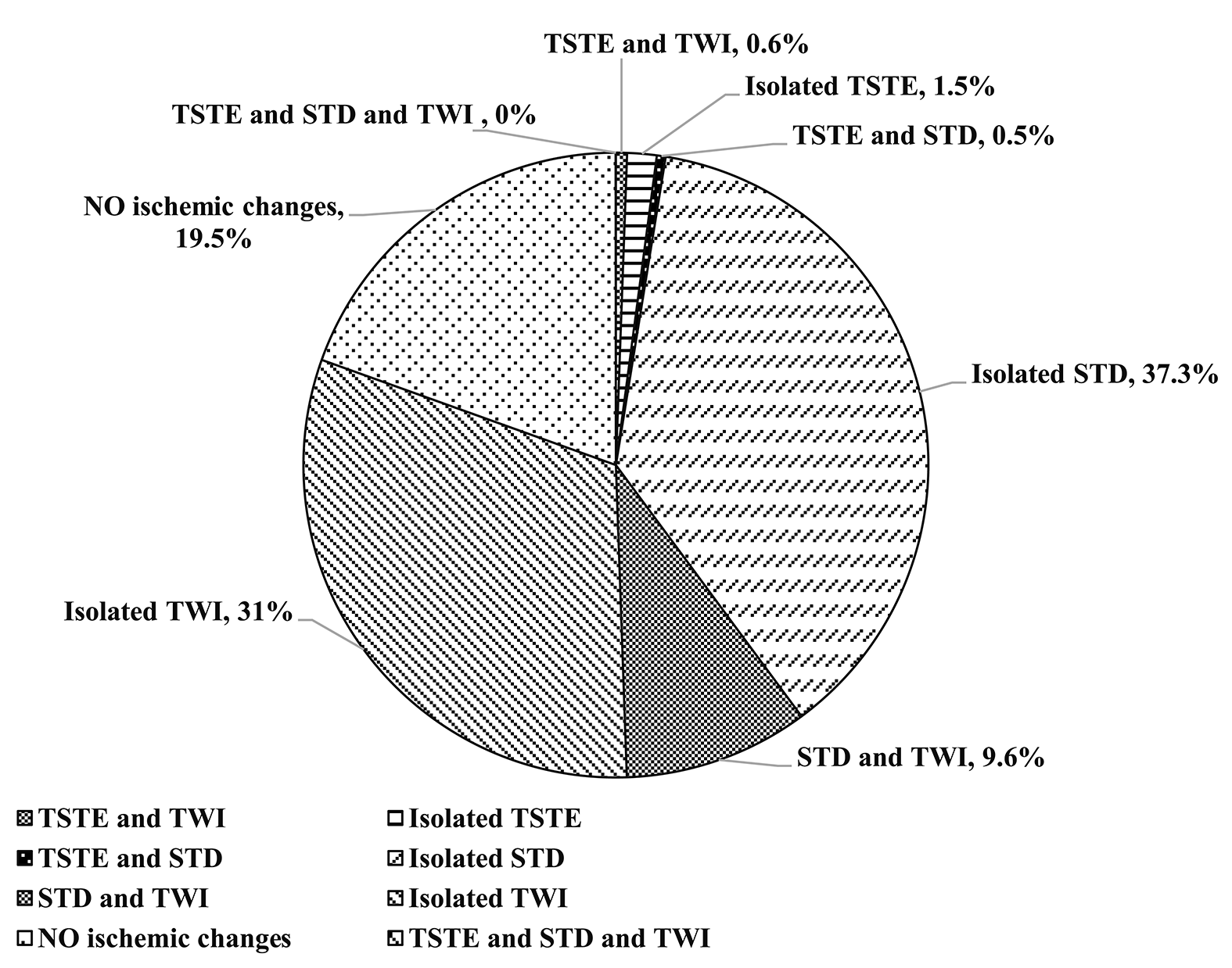

Supplement: Supplementary file 2 — Figure S1. Distribution of admission electrocardiographic findings. [file CLC-43-574-s002.tif]
